# Modelling cost-effectiveness of different vasectomy methods in India, Kenya, and Mexico

**DOI:** 10.1186/1478-7547-5-8

**Published:** 2007-07-13

**Authors:** Yancy Seamans, Claudia M Harner-Jay

**Affiliations:** 1 PATH, Seattle, WA, USA

## Abstract

**Background:**

Vasectomy is generally considered a safe and effective method of permanent contraception. The historical effectiveness of vasectomy has been questioned by recent research results indicating that the most commonly used method of vasectomy – simple ligation and excision (L and E) – appears to have a relatively high failure rate, with reported pregnancy rates as high as 4%. Updated methods such as fascial interposition (FI) and thermal cautery can lower the rate of failure but may require additional financial investments and may not be appropriate for low-resource clinics. In order to better compare the cost-effectiveness of these different vasectomy methods, we modelled the costs of different vasectomy methods using cost data collected in India, Kenya, and Mexico and effectiveness data from the latest published research.

**Methods:**

The costs associated with providing vasectomies were determined in each country through interviews with clinic staff. Costs collected were economic, direct, programme costs of fixed vasectomy services but did not include large capital expenses or general recurrent costs for the health care facility. Estimates of the time required to provide service were gained through interviews and training costs were based on the total costs of vasectomy training programmes in each country. Effectiveness data were obtained from recent published studies and comparative cost-effectiveness was determined using cost per couple years of protection (CYP).

**Results:**

In each country, the labour to provide the vasectomy and follow-up services accounts for the greatest portion of the overall cost. Because each country almost exclusively used one vasectomy method at all of the clinics included in the study, we modelled costs based on the additional material, labour, and training costs required in each country. Using a model of a robust vasectomy program, more effective methods such as FI and thermal cautery reduce the cost per CYP of a vasectomy by $0.08 – $0.55.

**Conclusion:**

Based on the results presented, more effective methods of vasectomy – including FI, thermal cautery, and thermal cautery combined with FI – are more cost-effective than L and E alone. Analysis shows that for a programme in which a minimum of 20 clients undergo vasectomies per month, the cost per CYP is reduced in all three countries by updated vasectomy methods.

## Background

Vasectomy is generally considered safe and effective for couples desiring a method of permanent contraception. The effectiveness of vasectomy, at one time thought to have an extremely low failure rate, has been called into question recently. According to recent research studies, the vasectomy technique that is most commonly used worldwide – simple ligation with excision (L and E) of a short segment of the vas – appears to have a relatively high failure rate, with reported pregnancy rates as high as 4% at the end of three years [1]. In order to lower the failure rate associated with LandE, updated vasectomy methodology will need to be adopted by practitioners worldwide. Currently used methods incorporating fascial interposition (FI) and/or thermal cautery could provide increased efficacy at costs appropriate for global settings.

Additional recent studies on the effectiveness of vasectomy have yielded updated data for L and E as well as the benefits of both FI and thermal cautery of the vas [2-4]. These high-quality studies indicate a failure rate (based on semen analysis) for L and E as high as 12.7%, while the addition of FI reduces the failure rate to 5%. Based on an observational trial, the failure rate while using cautery with excision is 0.8% (based on semen analysis) and may be further reduced with the addition of FI (quality data are still lacking). Additionally, retrospective country analyses have indicated failure as high as 4.2% to 8.7% (at the end of three years) in Nepal and China based on semen analysis or pregnancy outcomes [1,5].

The cost of providing vasectomies has been evaluated previously in several domestic and international settings, generally in relation to the "method mix" of different contraceptive methods [6-8]. Two studies that analysed the cost of providing contraceptive services in the United States estimated the cost of providing a vasectomy to be between approximately US$353 and US$756, depending on the payee [7,8]. Studies conducted internationally estimated the cost per vasectomy to be approximately US$103 in Iran, [6] US$298 in Mexico, [9] and US$29 in Zimbabwe [9].

Cost-effectiveness and cost-utility studies of vasectomy in relation to the contraceptive method mix have been conducted in multiple settings [6-8]. However, previous cost studies have not incorporated recently available information and have used overall estimated failure rates for vasectomy that ranged from 0.04% to 0.15% [6,8]. Similarly, Stover, et al. used a 0% failure in estimating the couple years of protection (CYP) for sterilisation [10]. Based on updated research [2-4,11] and estimates from retrospective country analyses of effectiveness, [1,5] these estimates may be significantly understated. Additionally, these previous studies have provided a general estimation of the cost-effectiveness of vasectomy but have not differentiated between different vasectomy methods and their differing effectiveness.

The purpose of this research was to model cost-effectiveness though an evaluation of different vasectomy methods used in clinics in Asia, Africa, and Latin America. To complete this evaluation, we conducted interviews at clinics in India, Kenya, and Mexico to better understand the cost of providing vasectomies. These cost data were then coupled with currently published effectiveness data in order to construct a cost-effectiveness profile for different vasectomy methods.

## Methods

### Study sites

We visited clinics in three countries and interviewed local personnel between February and June 2005. In order to best represent the diversity of current methods and clinic settings, we made an effort to conduct interviews within these countries at clinics that:

• Perform vasectomy using different methods.

• Perform vasectomy in urban areas and/or see a high number of patients per month.

• Perform vasectomy in rural areas and/or see a low number of patients per month.

Despite these general guidelines, clinics represented a convenience sample of facilities, and the sample was influenced by geographical proximity, permission from clinic managers and government officials, and recommendations from consultants or central health service staff.

### Data collection methodology

Initially, we collected background data and information from the Ministry of Health (MOH) and Government Statistics Office, nongovernmental organisations (NGOs), and clinics providing vasectomy services. We requested the following information:

• Types of vasectomy methods used.

• History of vasectomy programmes in country and region.

• Number of men per year getting vasectomies and the relationship in prevalence to other contraceptive methods.

• Observed/reported vasectomy effectiveness rates and the effectiveness rate in relationship to the vasectomy method used.

• Demographic and socioeconomic data on men/couples choosing vasectomy.

• Current salary rates, benefits, basic consulting rates, and other allowances for MOH personnel.

• MOH transport allowances such as transportation, per diem, etc.

• Drugs and supply lists and MOH essential drug lists.

• Information on health delivery systems for vasectomy from MOH, NGOs, and Social Security.

• Current or previous vasectomy research conducted in the country.

• Logistics of and impediments to introduction of new health technology into the country or region.

• Supply channels for clinic supplies and current issues regarding stock-outs or supply problems.

• Current training programmes for vasectomy services, integration of training into larger programmes, and the frequency of refresher training.

Secondly, we visited clinics providing vasectomies and interviewed health workers and administrators associated with vasectomy provision to understand all costs and activities for each of the different types of vasectomy procedures. Information gathered included:

• Monthly, weekly, and/or daily patterns of service delivery.

• Costs of materials/supplies, reprocessing, service delivery, and training.

• Service time for vasectomy activities.

• Supply/resupply patterns, channels, and responsible individuals.

• Fees for service.

• Personnel required for vasectomy services.

• Recruitment strategies for the target population and responsible individuals/organisations.

• Post-vasectomy follow-up protocol and treatment of complications.

• Current reporting forms used to monitor vasectomy patients and costs.

All data were collected based on interviews with clinic staff and not on direct observation of the vasectomy procedure. This allowed data collection during days in which there were no vasectomies – especially critical in both Kenya and India where the number of vasectomies can be very low on a daily basis. However, this limited the information to anecdotal reporting on the duration of vasectomy services.

### Cost collection parameters

The evaluation was developed in order to collect the following types of cost information:

• Economic costs. Costs are inclusive of all resources regardless of who provided them (e.g., donors, central procurement, local procurement) [12].

• Programme costs. Costs include associated costs to the programme and/or clinic but not those costs borne by the clients. Clients' time in the clinic, travel expenses, lost work time, or other costs were not included in this evaluation.

• Costs from fixed vasectomy service programmes. No mobile services or vasectomy camp costs were evaluated.

• Direct costs. No indirect costs (e.g., administrative costs, programme, or hospital administration salaries) were included in the evaluation.

• Both joint and nonjoint costs were included. Joint costs are items used for multiple clients (e.g., no scalpel vasectomy [NSV] kits, staff salaries) and nonjoint items are used for a single client (e.g., gauze).

• Both recurrent and capital costs were included for supplies and materials. However, capital costs for the building and building supplies (e.g., tables, chairs) that were not limited to use with vasectomy were not included. Recurrent costs such as electricity, lighting, and general supplies were also not included.

### Overall assumptions

Assumptions were made for each clinic in each of the sites. While some assumptions are country specific, the following general assumptions were made:

1. Annualisation versus simple depreciation. Despite a general precedent in cost analyses for doing so, capital costs were not annualised. Most items were less than US$100 (e.g., decontamination containers, NSV kits) and do not merit annualisation [12]. For items with costs greater than US$100 (e.g., autoclaves), the decision was made to evaluate the costs based on a simple depreciation model because this equipment was necessary for the proper operation of the clinic and therefore did not carry a large opportunity cost.

2. The rate of vasectomy services remains the same over time for a specific clinic/doctor.

3. Materials and equipment purchase prices are based on current prices when available.

4. In the case of missing cost data, costs were estimated using similar clinics (in the same country and/or under the same service system).

5. Hazardous waste disposal costs were not included since vasectomy represented a very small portion of the waste stream, and accurate estimates were not possible.

6. Monthly salaries were converted to annual salaries by multiplying by 12. Vacation benefits were accounted for by dividing the annual salary by the actual number of days worked annually. Other benefits (e.g., fringe benefits, travel allowances) were not included in the cost model.

7. If the actual lifespan of equipment was unknown, it was assumed that NSV equipment, surgical gowns, and surgical cloths were usable for five years.

8. If NSV equipment was donated, its cost was based on costs from an Indian manufacturer (forceps and clamp). However, the costs associated with procurement and import of this equipment were not included in the overall cost summary.

9. For supplies that are used with multiple patients (joint supplies) and that are not limited to use with vasectomies (e.g., generic washing basin), use was generally estimated as a percentage of the overall use. In clinics with a limited number of clients, it was assumed that a vasectomy would account for one day of use and that the overall use rate of the item was 75% of available clinic days (e.g., a basin was used 15 out of 20 weekdays in a month).

10. Depending on the number of vasectomies given at a clinic, certain equipment was included or not included. For example, if a clinic performed one vasectomy per year but used its autoclave on a daily basis for other procedures, the cost of including usage for a vasectomy into the usual use was considered negligible.

### Currency conversion

Costs were collected in local currency and converted to US dollars using mid-market rates (as of June 10, 2005). Conversion rates for US$1 were as follows:

• 76.7000 Kenyan shillings

• 43.5150 Indian rupees

• 10.8686 Mexican pesos

All costs in the text and tables are listed in US dollars.

### Calculating cost estimates

Mean vasectomy provision costs for each country were calculated for each type of service cost (e.g., vasectomy materials, service labour, reprocessing materials) from the data directly collected at each clinic. Training costs were calculated by determining the cost to conduct in-country training and educate an individual doctor and then amortising the cost over the number of vasectomies that he or she could expect to perform in the next five to ten years. This produced a range of cost per vasectomy that was averaged to provide an indicative training cost per doctor for a given country.

### Cost estimates for fascial interposition and thermal cautery

Because diversity of vasectomy methods was limited in a given country (as described in Results) and often limited to simple L and E, we estimated costs for different vasectomy methods based on the current cost of performing a vasectomy in a specific country, plus the additional costs of adding an additional method, including training costs. Average costs were used when cost ranges existed for individual cost components. For India and Kenya, the "base" cost method was simple L and E since it is the method most commonly used at the clinics studied. In Mexico, the "base" cost was L and E with FI, since it is the most commonly used method in the clinics where we conducted interviews. A cost estimate for simple L and E in Mexico was calculated by subtracting the additional time required for FI from the base cost.

#### Estimated additional cost for fascial interposition

The cost of introducing FI is limited to the cost of training or retraining service providers and the additional time required to perform the vasectomy operation. Training costs vary depending both on the specific country situation and on the extent of retraining necessary. Additional labour costs are limited to the time required to perform the FI during the vasectomy operation. The randomised control trial conducted by Family Health International (FHI) [4] estimated the additional time at two to three minutes. This will add the average labour cost of two minutes of a doctor's salary to each operation.

#### Estimated additional cost for thermal cautery

The cost of introducing thermal cautery includes the cost of training or retraining service providers, the material cost for the cautery device and a reusable handpiece sleeve, the additional time required to perform the vasectomy operation, and the additional time required to reprocess the equipment.

Training costs were estimated as indicated above plus additional costs associated with procuring thermal cautery devices for the training.

Materials costs were estimated using current costs of Aaron/Bovie thermal cautery devices. Cautery handpieces cost approximately US$37.40, and tips cost approximately US$3.85. If costs are amortised over 200 uses for the handpiece and 20 uses for the tips, the total additional cost per vasectomy is approximately US$0.38. Additionally, the cost of two high-quality alkaline AA batteries should be included and will vary depending on the location. Batteries are estimated to last for approximately 20 procedures based on a previous PATH evaluation (unpublished). These material costs do not include the cost of importation or the difficulty of introducing a nondomestic device into the medical equipment procurement system.

The cost of a reusable and sterilisable cloth handpiece sleeve was estimated at US$0.50 and the cost was amortised over 100 uses, leading to an additional cost per vasectomy of US$0.005.

The additional time required to perform the vasectomy with thermal cautery in comparison to simple L and E has not been rigorously studied. Anecdotal evidence from Labrecque suggests that no extra time is required to perform occlusion with cautery in comparison to ligation with suture (personal communication).

The additional time required to reprocess the equipment is limited to the time required for cleaning the tip. Since the tips can be decontaminated and disinfected with the rest of the equipment in bleach or glutaraldehyde, there is not a significant additional cost component associated with these stages of reprocessing. If cleaning time is estimated at one minute, the additional cost will be based on an average nurse's salary for this duration.

### Effectiveness data

As shown in Table 1, effectiveness data from the recent studies were used to estimate the rate of severe oligospermia at 12 and 24 weeks (or 26 weeks, depending on the study) after the vasectomy [2-4]. Vasectomy failures at 24 or 26 weeks were considered overall failures and the man was considered virile. Confidence intervals from the literature were used to estimate ranges of effectiveness in order to provide a sensitivity analysis. Because of the lack of effectiveness evidence for thermal cautery with FI, an estimate was made that exceeded the effectiveness of cautery or FI alone. The range of effectiveness for cautery with FI was based on a binomial distribution (as used in the literature for the other methods).

**Table 1:** Effectiveness data for all age groups (effectiveness range)

Method	Proportion Oligospermic at 12 Weeks	Proportion Oligospermic at 24 Weeks	Proportion Virile at 24 weeks
L and E*	0.82 (0.78 – 0.86)	0.873 (0.84 – 0.91)	0.127 (0.9 – 0.16)
L and E with FI*	0.91 (0.88 – 0.94)	0.952 (0.926 – 0.97)	0.048 (0.03 – 0.074)
Cautery^γ^	0.964 (0.94 – 0.981)	0.99 (0.977 – 0.998)	0.01 (0.002 – 0.023)
Cautery with FI^ψ^	0.97 (0.947 – 0.984)	0.995 (0.982 – 0.999)	0.005 (0.001 – 0.018)

* Based on data from Sokal D, Irsula B, Hays M, Chen-Mok M, Barone MA: Vasectomy by ligation and excision, with or without fascial interposition: a randomized controlled trial [ISRCTN77781689]. *BMC Medicine *2004, 2:6.

^γ ^Based on data from Barone MA, Irsula B, Chen-Mok M, Sokal DC: Effectiveness of vasectomy using cautery. *BMC Urology *2004, 4:10 *and *Sokal D, Irsula B, Chen-Mok M, Labrecque M, Barone MA: A comparison of vas occlusion techniques: cautery more effective than ligation and excision with fascial interposition. *BMC Urology *2004, 4:12.

^ψ ^Based on estimation from above studies using a binomial distribution as the confidence interval.

### Calculating cost-effectiveness

We based our calculations of cost-effectiveness on the metric of CYP. CYP is the estimated period of protection from conception for a family planning method [12] and is a standard benchmark that permits comparison of different family planning methods (e.g., injectable contraceptives, intrauterine devices, condoms). Standard values for CYP for vasectomy have been empirically estimated for the three countries included in this evaluation by Stover, et al. (India-13 CYP, Kenya-9 CYP, Mexico-8 CYP) [10]. The differences in CYP values between countries reflect the age at which the man would typically be vasectomised. For this evaluation, adjusted CYP was calculated by multiplying the CYP literature values by the percentage of effectiveness at 24 weeks (as defined by severe oligospermia). Cost-effectiveness was then calculated as cost per CYP by dividing the average estimated cost by the adjusted CYP.

## Results

### Clinic information

Data was collected from 21 clinics in India, Kenya, and Mexico. Focus was placed on public clinics (e.g., Ministry of Health, Family Planning Associations of India and Kenya) in lieu of private facilities. In general, each clinic performed one type of vasectomy exclusively – the clinics in India and Kenya typically provided vasectomies using L and E while the majority of clinics in Mexico used FI. None of the clinics interviewed used thermal cautery. The frequency of vasectomy service varied widely between countries and individual clinics – ranging from less than one per month in some clinics in Kenya and India to 27 per month in Mexico. Additional clinic information is found in Table 2.

**Table 2:** Comparative clinic data

	India	Kenya	Mexico
Number of clinics included in study	5	10	6
Type(s) of clinics	Family Planning Association of India (FPAI)	MOH, Family Planning Association of Kenya (FPAK), Marie Stopes International	IMSS (Social Security), MOH
Location of clinics	Urban	Urban/town	Urban/rural
Number of staff	14–37	4–9 (clinics), unknown (district hospitals)	300–400 (urban)
Number of doctors	1–4	1 (clinic), unknown (district hospitals)	55–85 (urban), 7 (rural)
Type(s) of vasectomies provided	L and E (4/5 NSV, 1/5 incisional)	L and E (including one using incisional), two FI	L and E (2/6), FI (4/6)
Number of vasectomies	Between 4/year and 6/month	0–11/year	15–27/month (urban), 2/month (rural)

### Vasectomy cost estimates with current methods

Costs for performing vasectomies varied greatly from country to country and at different clinics within one country. Average vasectomy service costs in each country are $18.17 in India, $52.89 in Kenya, and $38.73 in Mexico (Table 3). In the case of Kenya, a representative rather than an average cost is presented due to the scarcity of data available during the interviews. The greatest cost component in each country (not including training in Kenya) is labour. This is especially true in Mexico, where the labour rates are higher in comparison to the other countries. Labour costs were calculated based on salary information obtained from service providers. Duties vary by location and can be summarised as follows:

**Table 3:** Summary of baseline cost data by country and vasectomy method

	L and E		L and E		L and E with FI	
Item	Average Cost	Percent	Representative Cost	Percent	Average Cost	Percent

Vasectomy Materials (supplies and equipment)	$3.83	21%	$5.05	10%	$2.64	7%
Service labour (vasectomy plus follow-up)	$6.76	37%	$11.67	22%	$28.12	73%
Reprocessing materials	$1.18	7%	$0.42	<1%	$0.80	2%
Reprocessing labour	$2.77	15%	$1.94	4%	$2.72	7%
Follow-up materials/patient supplies	$3.37	19%	Unknown	N/A	$4.24	11%
Subtotal without training	$17.91		$19.08		$38.52	
Training (without materials)	$0.26	1%	$33.81	64%	$0.21	<1%
Total including training	$18.17		$52.89		$38.73	

• Patient intake is performed by receptionists, social workers, medical assistants, and doctors (in smaller clinics).

• Pre-vasectomy counselling is performed by social workers, nurses, and doctors.

• Operating room preparation is done by nurses, ANMs (auxiliary nurse midwives in India), nursing assistants, and male attendants.

• Patient preparation is performed by male attendants and nurses.

• Vasectomies are performed by doctors, sometimes assisted by nurses.

• Operating room cleanup is performed by cleaning services, ANMs and attendants, nurses, nursing assistants, and male attendants.

• Post surgery counselling is performed by doctors, counsellors, and nurses.

Training costs varied from country to country depending on the base cost associated with supplies and materials, recruiting model patients, travel expenses, and the number of patients that the doctor will see during the subsequent five to ten years after the training. Training costs in Kenya are drastically different than in other sites due to the low volume of vasectomy patients and the higher cost of amortising the training cost over this number of patients.

#### Additional costs for fascial interposition and thermal cautery

As described in Methods, cost estimations were calculated to represent the additional costs of performing vasectomy with FI and thermal cautery. Additional estimated cost per vasectomy for these methods in India and Mexico ranged from $0.37 to $0.75, representing a small fraction of the base cost of performing a vasectomy. In Kenya, additional estimated costs were significantly higher, up to $34.36, because of the significant impact of training expenditures on the overall cost. Additional costs for each country including contributions due to time, training, and materials are listed in Table 4.

**Table 4:** Summary of additional per-vasectomy cost using FI and cautery in each country

	Time	Training	Materials	Estimated Total (as average of individual costs)
Fascial Interposition				

India	$0.08 – $0.13	$0.04 – $0.48	N/A	$0.37
Kenya	$0.24 – $0.28	$8.82 – $58.80	N/A	$34.07
Mexico	$0.33 – $0.66	$0.11 – $0.30	N/A	$0.71

Cautery				

India	$0.01	$0.04 – $0.48	$0.42	$0.69
Kenya	$0.04	$8.82 – $58.80	$0.51	$34.36
Mexico	$0.08	$0.11 – $0.30	$0.46	$0.75

### Cost-effectiveness of different vasectomy methods

In order to determine the cost-effectiveness of alternative interventions and to best inform policy choices for a country or region, an active vasectomy programme should be considered. In Mexico, where clinics often perform more than 20 vasectomies per month, the system can be considered adequately robust to distinguish the cost-effectiveness between different methods. However, in India and Kenya, where the vasectomy rates are much lower, the current costs do not represent the costs of a programme functioning at its full capacity. Therefore, vasectomy cost estimates for both India and Kenya were modified to represent an approximate monthly client load of 20. We believe that this will better represent the cost-effectiveness of different vasectomy methods of an active and sustainable vasectomy programme.

#### Modified cost data

Cost data from Mexico was used without adjustment. However, as noted above, costs from both India and Kenya were modified to approximate a programme that performed 20 vasectomies monthly at each clinic. Increasing the monthly rate of vasectomies lowered the base cost of performing a vasectomy with L and E by 5% and 13% in India and Kenya, respectively. While the modelled increase in clients was considerable in both cases, the overall reduction in cost is not more dramatic since the labour required to perform the vasectomy remains the same. Some cost saving is achieved through a greater utilisation of equipment during its lifetime and by assuming that the facility batches instruments from multiple vasectomies during reprocessing (e.g., washing and sterilising two sets of NSV tools at the same time). A drastic reduction in training costs was observed in Kenya with a final estimated cost of approximately $0.55 per vasectomy, amortised over a time period of five to ten years. Both original and modified cost data is summarized in Table 5.

**Table 5:** Summary of country-specific total per-vasectomy cost using different methods using original data and modified based on a more robust vasectomy program

Original Data				
	L and E	L and E with FI	Cautery	Cautery with FI

India	$17.91	$18.28	$18.60	$18.71
Kenya	$19.08	$53.15	$53.35	$53.61
Mexico	$37.82	$38.52	$38.57	$39.07

Modified Data				

	L and E	L and E with FI	Cautery	Cautery with FI

India	$16.59	$16.72	$17.04	$17.15
Kenya	$16.52	$17.33	$17.62	$17.88
Mexico	$37.82	$38.52	$38.57	$39.07

#### Cost per couple year of protection

The cost per CYP is modestly reduced in all three countries by selection of a more effective vasectomy method than L and E. By choosing FI, thermal cautery, or cautery with FI, the cost per CYP is reduced by $0.11 – $0.14 in India, $0.08 – $0.12 in Kenya, and $0.36 – $0.55 in Mexico (Table 6). In each case, the cost per CYP is minimized by choosing cautery without adding FI.

**Table 6:** Vasectomy cost per adjusted CYP and incremental cost per CYP in India, Kenya, and Mexico

	Adjusted CYP	Adjusted CYP Range	Cost per Adjusted CYP	Cost per CYP Range
India				

L and E	11.349	10.92 – 11.83	$1.46	$1.40 – $1.52
L and E with FI	12.376	12.038 – 12.61	$1.35	$1.33 – $1.39
Cautery	12.87	12.701 – 12.974	$1.32	$1.31 – $1.34
Cautery with FI	12.935	12.766 – 12.987	$1.33	$1.32 – $1.34

Kenya				

L and E	7.857	7.56 – 8.19	$2.10	$2.02 – $2.19
L and E with FI	8.568	8.334 – 8.73	$2.02	$1.99 – $2.08
Cautery	8.91	8.793 – 8.982	$1.98	$1.96 – $2.00
Cautery with FI	8.955	8.838 – 8.991	$2.00	$1.99 – $2.02

Mexico				

L and E	6.984	6.72 – 7.28	$5.42	$5.20 – $5.63
L and E with FI	7.616	7.408 – 7.76	$5.06	$4.96 – $5.20
Cautery	7.92	7.816 – 7.984	$4.87	$4.83 – $4.93
Cautery with FI	7.96	7.856 – 7.992	$4.91	$4.89 – $4.97

## Discussion

### Cost of labour to perform vasectomy

Labour has been noted as the greatest cost in previous studies of family planning services [9] Similarly, in all sites in this study, especially in Mexico, staff labour during counselling, service delivery, and instrument reprocessing accounted for a significant portion or a majority of the overall cost of providing a vasectomy. However, the actual vasectomy procedure does not represent a majority of the labour time. This should be considered when discussing an intervention that has an impact on the time required for the vasectomy but improves the overall effectiveness. While a more complicated method may represent a significant increase in time in comparison to the discrete time for a simpler method (e.g., 2 extra minutes to perform FI in addition to the 12 minutes for L and E), it may not be as significant when regarding the entire required labour (including pre- and post-vasectomy counselling, reprocessing, and semen analysis).

### Cost of training

In Kenya, additional cost savings would be realised through lowering training costs that occur with a greater acceptance of vasectomy. Current training costs include the recruitment of patients as a significant portion of the overall training cost. With a more robust vasectomy programme, media promotion could be reduced or eliminated.

### Rate of failure and complications based on vasectomy frequency

In a survey of urologists in the United States, the reported incidence of vasectomy failure among practitioners who performed more than 12 vasectomies in the last year was half that of practitioners performing fewer procedures [13]. Additionally, evidence from a systematic MEDLINE review indicates that a low number of vasectomies being performed annually has implications for a greater number of side effects [14]. This review of safety and effectiveness of vasectomy indicates a 1.6% to 4.6% rate of hematoma and a 3.5% incidence of infection. Differences in the rates of hematoma among doctors corresponded to the number of vasectomies performed annually and was not related to the method of vas occlusion.

These results have implications for patients receiving vasectomies in clinics that perform very few vasectomies per year, as seen in India and Kenya. Actual effectiveness may vary greatly in these countries from data that is based on vasectomies provided by experienced practitioners who perform vasectomies routinely (as presented in this report). Additionally, patients in these countries may experience a higher-than-expected rate of undesirable complications that may impact their acceptance and personal promotion of vasectomy.

### Social impact of vasectomy failure

While not quantified in this study, there is a definite social impact associated with vasectomy failure. Despite appropriate counselling on the possibility of failure and the need for post-vasectomy semen analysis, many patients do not return for analysis and risk the possibility of an undiagnosed failure and possible future conception. This can affect both the couple, due to questions regarding infidelity, and the vasectomy program in general as vasectomy may then be viewed in the community as an ineffective method. Because of this, there may be additional reasons besides cost-effectiveness to choose a more effective vasectomy method.

### Study limitations

Because of limited data on vasectomy complications associated with different methods, the cost associated with potential complications was not included in the analysis. No data on complications were collected from the clinics.

Because large-scale capital costs were not included, the costs presented may underestimate the cost of maintaining a vasectomy programme. Costs presented represent the cost of providing a vasectomy but do not include the opportunity costs of the real estate, maintenance of the infrastructure (e.g., building, lights, and furniture), or additional capital costs associated with a vasectomy programme. Capital costs have been eliminated from at least one other reproductive health service cost analysis [9]. The reasons cited include lack of good information on governmental services, sensitivity to assumptions, and projected low marginal costs for adding additional services due to clinics not operating at full capacity. As noted in the background, cost estimates for vasectomies in other countries have varied widely. These differences are likely due to differences in costing methodology that may or may not include additional capital costs and full representation of training, represent private rather than public clinic services, and employ different assumptions in estimating costs that were not directly collected.

Most operational times were estimated by the staff to the nearest five minutes during the interviews which may inaccurately reflect the actual time spent on activities, especially those of short duration. Additionally, non-client time was not included and this may undervalue the amount of time related to vasectomy services that is not spent in direct contact with the patient.

Vasectomy services in India were limited to fixed clinics based on the suggestion of MOH staff. However, it has been reported (David Sokal, FHI, personal communication, August 2005) that mobile camps provide a significant portion of the vasectomies in India. Mobile camps could have an entirely different cost structure from fixed facilities and could have significantly lower initial counselling and follow-up costs while incurring additional costs for transportation of equipment and personnel.

## Conclusion

Based on the results presented, more effective methods of vasectomy, including FI, thermal cautery, and thermal cautery combined with FI, are more cost-effective than Land E alone. Methods with higher effectiveness, although they increase the incremental cost of providing a vasectomy, still reduce the number of unintended pregnancies after vasectomy and may provide additional benefits to vasectomy programmes.

Introduction of FI and thermal cautery into existing vasectomy programmes and trainings should occur when possible in order to maximise the cost-effectiveness of ongoing programmes. New trainings should incorporate these methods when possible in order to establish the most cost-effective country vasectomy programme.

## Abbreviations

ANM Auxiliary nurse midwives

CYP Couple years of protection

FI Fascial interposition

L and E Ligation and excision

MOH Ministry of Health

NGO Nongovernmental organisation

NSV No scalpel vasectomy

## Competing interests

The author(s) declare that they have no competing interests.

## Authors' contributions

CHJ participated in the design of the cost model, organisation of the data collection, and cost analysis. YS participated in the design of the cost model, data collection, cost analysis, and drafted the manuscript. Both authors read and approved the final manuscript.
